# A Study of Brain Networks Associated with Swallowing Using Graph-Theoretical Approaches

**DOI:** 10.1371/journal.pone.0073577

**Published:** 2013-08-29

**Authors:** Bo Luan, Peter Sörös, Ervin Sejdić

**Affiliations:** 1 Department of Electrical and Computer Engineering, Swanson School of Engineering, University of Pittsburgh, Pittsburgh, Pennsylvania, United States of America; 2 Department of Clinical Neurological Sciences and the School of Communication Sciences, Western University, London, Ontario, Canada; Beijing Normal University, Beijing, China

## Abstract

Functional connectivity between brain regions during swallowing tasks is still not well understood. Understanding these complex interactions is of great interest from both a scientific and a clinical perspective. In this study, functional magnetic resonance imaging (fMRI) was utilized to study brain functional networks during voluntary saliva swallowing in twenty-two adult healthy subjects (all females, 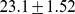 years of age). To construct these functional connections, we computed mean partial correlation matrices over ninety brain regions for each participant. Two regions were determined to be functionally connected if their correlation was above a certain threshold. These correlation matrices were then analyzed using graph-theoretical approaches. In particular, we considered several network measures for the whole brain and for swallowing-related brain regions. The results have shown that significant pairwise functional connections were, mostly, either local and intra-hemispheric or symmetrically inter-hemispheric. Furthermore, we showed that all human brain functional network, although varying in some degree, had typical small-world properties as compared to regular networks and random networks. These properties allow information transfer within the network at a relatively high efficiency. Swallowing-related brain regions also had higher values for some of the network measures in comparison to when these measures were calculated for the whole brain. The current results warrant further investigation of graph-theoretical approaches as a potential tool for understanding the neural basis of dysphagia.

## Introduction

Dysphagia (swallowing difficulties) may arise from the entry of foreign matter into respiratory pathways [1]. It is a serious condition that often accompanies acute stroke, acquired brain damage, and neuro-degenerative illnesses [2]. Patients with swallowing difficulties are vulnerable to the entry of foreign matter into the respiratory tract. This foreign matter will greatly increases the occurrence of severe respiratory problems among dysphagia patients. Therefore, understanding the neural basis of dysphagia is one of the paramount steps needed to develop future rehabilitation procedures.

The human brain is considered to be a large-scale robust and interactive biological system with non-trivial topological properties [3], such as hierarchy and small-world properties [4]. The human brain is considered to be one of the most complex networks found in nature. This biological system responds to external stimuli by transporting signals between specialized brain regions. Therefore, the study of brain functional connectivity contributes greatly to the understanding of brain functions and pathology.

Previous studies on graph theory suggested the possibility of performing network analysis on the human brain [4]. Using network analysis, the large variability of the brain structure could be abstractly reduced to a collection of nodes and links (edges). For functional networks, brain regions are represented by nodes and connections between regions are represented by links. By utilizing graph-theoretical approaches, the differences and similarities in the structure of brain functional networks can be easily identified. Also, the brain network shows consistent topology so that properties, such as small-worldness, could generally be identified in all human brain networks [5]. Furthermore, given that network nodes stand for brain regions and links stand for connections between them, comparison between different kinds of networks become fairly feasible [5].

Recent neuroimaging studies have consistently demonstrated evidence that swallowing is associated with activation in multiple regions of the human brain [6], [7], [8], [9], [10], [11], [12], [13], [14]. Previous analyses of brain functions during swallowing revealed activation clusters in the supplementary motor area, anterior cingulate and paracingulate gyri, pre- and postcentral gyrus [15]. Several other regions have also been found related to swallowing, including the posterior insula [16], basal ganglia, thalamus, and cerebellum. Despite these findings, interactions between different swallowing-related brain regions are still not well understood. Therefore, the study of brain functional connectivity during swallowing will contribute greatly to the understanding of brain integration and segregation. To accomplish this task, previous studies suggested graph theory as a valuable tool for performing network analysis on human brain neuroimaging studies [4], [5]. The graph-theoretical approaches enable us to accomplish a comparison between different kinds of networks [5].

The goal of this study is to use graph-theoretic approaches to examine the interaction between brain regions during voluntary saliva swallowing in healthy young adults and compare network properties between and within subjects. To be specific, we aim to determine inter- and intra- hemispherical connections during swallowing tasks. Furthermore, differences between the whole-brain network and various regions of interest (ROIs) on computed network measures will be studied.

## Materials and Methods

### Data Acquisition

Twenty-two healthy young-adult subjects, all females (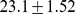 years), participated in this study after providing written, informed consent. The study protocol was approved by the University of South Carolina Institutional Review Board.

All functional magnetic resonance scans of the brain were acquired on a Siemens Magnetom Tesla Trio Tim scanner with a 32-channel RF-receive head coil at the McCausland Center for Brain Imaging, University of South Carolina, Columbia, SC, USA. These blood oxygen level dependent (BOLD) images were acquired using an echo planar imaging sequence in 36 axial slices (TR = 2200 ms, TE = 35 ms, flip angle  = 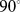, FOV = 192 mm, 3 mm thickness) during swallowing. During our experiment, participants were instructed to swallow their accumulated saliva every 44 seconds (every 20 volumes acquired). They were directed to move as little as possible. They were also instructed not to produce exaggerated oral movements to increase or manipulate the accumulation of saliva. The saliva should be accumulated passively prior to swallowing. A comfortable custom-built restraint was applied during fMRI scans to minimize head movement. A high-resolution T1-weighted MRI sequence was also performed during the data collection (3D MP-RAGE, 176 axial slices with 1 mm slice thickness, a 256 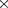 256 matrix, and 256 mm 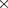 256 mm FOV).

### Data Preprocessing Steps

#### fMRI Data Preprocessing

All data in the study were preprocessed using Statistical Parametric Mapping (SPM) software [17]. For each subject 350 volumes of the scans were acquired, and the first 10 scans were discarded for magnetic equilibrium. The remaining each of the 340 volumes underwent the following four preprocessing steps sequentially: realignment, coregistration, normalization, and smoothing. Excess motion defined as greater than 4.0 mm of translation/rotation was eliminated in any of the task-free scans.

Specifically, the fMRI scans for each subject were first adjusted for time delay between different scans. Second, for each subject the images were realigned to the first slices among all slices using a least squares fitting algorithm and a 6 parameter rigid body transformation [18] to correct for head motion. The following formula for head movement calculates the group difference in translation and rotation [19]:
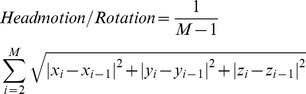
(1)where 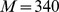 represents the length of the time series. The 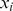, 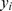 and 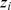 are the translations or rotations magnitude in the 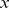, 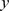 and 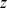 directions at 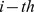 time point, respectively.

After removing the movement interference in fMRI images, the fMRI images further underwent the coregistration step during which the mean fMRI scans were overlayed on a high resolution anatomical image to maximize the mutual information. Therefore, all other functional images were resliced to align with the reference image.

Then, to make inter-individual comparisons, normalization was then performed to warp the images to fit a standard MNI (Montreal Neurological Institute) template. Finally, smoothing was applied with Gaussian kernel with a 4-mm full-width at half maximum to suppress noise and effects due to residual differences [17].

#### Anatomic Parcellation

The choice of nodes and links greatly influences the results of network connectivity analysis [20]. We chose the parcellation (segmentation) scheme that has been used previously in many network studies (e.g., [21], [19], [22], [23]). Therefore, the preprocessed fMRI datasets were parcellated into 116 anatomical ROIs via the automated anatomical labeling (AAL) template [24]. The AAL parcellation scheme segments the cerebrum into 90 cortical and subcortical anatomical ROIs (45 ROIs in each hemisphere) [24]. It divides the cerebellum into 26 ROIs (8 in the vermis and 18 in the cerebellar hemisphere, 9 in each side of the cerebellar hemisphere). This study considered the 90 cerebrum regions summarized in Table 1. This parcellation scheme provides non-overlapping segmentation of the entire brain volume such that each brain area depicted in AAL only points to one brain region in Table 1. These individual anatomical ROIs were parcellated from the whole brain by the MarsBaR toolbox [25]. Therefore, for each subject, we generated 90 time series for all the 90 anatomical ROIs in Table 1. The mean time series is the average of voxels for every time point in the time series over all 22 subjects in the study. This procedure generated the mean time series with 340 time points. These 90 mean time series were then correlated with each other to establish a 90 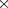 90 brain functional connectivity matrix.

**Table 1:** Cortical and sub-cortical regions (45 in each cerebral hemisphere; 90 in total) as anatomically defined in the AAL template and their corresponding abbreviations used in this study.

Region	Abbreviation	Region	Abbreviation
Precentral gyrus	PreCG	Supramarginal gyrus	SMG
Postcentral gyrus	PosCG	Precuneus	PCUN
Rolandic operculum	ROL	Superior occipital gyrus	SOG
Superior frontal gyrus, dorsolateral	SFGdor	Middle occipital gyrus	MOG
Middle frontal gyrus	MFG	Inferior occipital gyrus	IOG
Inferior frontal gyrus, opercular part	IFGoper	Cuneus	CUN
Inferior frontal gyrus, triangular part	IFGtri	Calcarine fissure and surrounding cortex	CAL
Superior frontal gyrus, medial	SFGmed	Lingual gyrus	LING
Supplementary motor area	SMA	Fusiform gyrus	FFG
Paracentral lobule	PCL	Temporal pole: superior temporal gyrus	TPOstg
Superior frontal gyrus, orbital part	SFGorb	Temporal pole: middle temporal gyrus	TPO
Superior frontal gyrus, medial orbital	SFGmedorb	Anterior cingulate and paracingulate gyri	ACP
Middle frontal gyrus, orbital part	MFGorb	Median cingulate and paracingulate gyri	MCP
Inferior frontal gyrus, orbital part	IFGorb	Posterior cingulate gyrus	PCG
Gyrus rectus	GRE	Hippocampus	HIP
Olfactory cortex	OLF	Parahippocampal gyrus	PHG
Superior temporal gyrus	STG	Insula	INS
Heschl gyrus	HES	Amygdala	AMY
Middle temporal gyrus	MTG	Caudate nucleus	CAU
Inferior temporal gyrus	ITG	Lenticular nucleus, putamen	PUT
Superior parietal gyrus	SPG	Lenticular nucleus, pallidum	PAL
Inferior parietal, but supramarginal and angular gyri	IPL	Thalamus	THA
Angular gyrus	ANG		

### Graph Theory Analysis

Graphs are sets of nodes and links. Nodes are the most basic element in functional network analysis. Links can be used as undirected paths, meaning that it can go both directions. Links can also be directed, meaning a node can traverse the network either forward or backward but never reverse direction [5]. For brain functional networks, nodes may represent neurons, cortical areas or brain regions whereas links may represent correlations. Therefore, links could depict activity patterns between nodes and form functional connectivity among nodes [5].

#### Network Measures

Using a graph-theoretical definition, a network is a collection of sets of nodes and links, where a node is considered as the most essential element of the network [20]. A graph theory based approach can quantitatively and analytically depict a wide variety of measures for brain networks. However, various measurements can describe a network in an effective way. Therefore, only some of the measurements that were used in previous connectivity studies are discussed here.

For binary undirected networks, we use 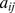 to represent the connection status in the network between node 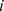 and 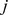. 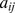 = 0 when no connection exists between two nodes and 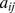 = 1 when the connection is present between two nodes. For weighted undirected networks, 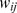 is the connection between nodes 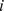 and 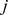, and it has range 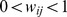. Because of the limitation of current fMRI neuroimaging techniques, the weighted directed network cannot be constructed in this study.

The node degree describes the number of direct connections a node has with the rest of the nodes in the network. The node degree is considered to be the most fundamental network measure. It is also a foundation for most of the network measures in this study. The summation of all the node degrees in a set in the network derives a degree distribution [26]. In a random network, connections are distributed randomly and uniformly with a symmetrical Gaussian shape and centered degree distribution [27]. A brain functional network, however, has a non-Gaussian distribution with a tendency to spread towards higher degrees [26]. Thus, we later introduce the rank-sum test to discuss the difference between two different groups.

The degree 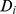 of a node 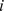 is the number of nodes directly connected to the 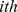 node. For a binary network, the node degree is defined as 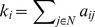 and for a weighted network it is defined as 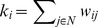, where 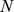 is the set of all nodes in a collection, and 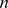 is the number of nodes in the collection. Given that the whole brain was parcellated into 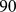 ROIs, therefore, 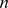 is equal to 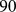, and 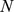 is the set of different possibilities (e.g., 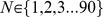. The degree of the entire network, therefore, is calculated by averaging all the nodes in the network:
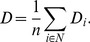
(2)


The clustering coefficient 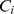 of a node 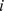 calculates the ratio between the number of existing connections and the maximum number of connections in a set of nodes [28]. The existing connections here are defined as the links between the direct neighbors of the node 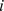. Connections in random networks are uniformly and randomly distributed so that clustering coefficients are relatively low for a random network, whereas complex networks contain densely connected clusters leading to a higher clustering coefficient [27]. For a binary network, the clustering coefficient 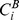 of the node 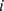 is calculated as [29]:
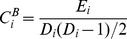
(3)in which 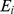 is the number of links in 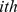 set of nodes 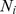 (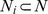), and 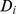 is the degree of node 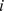 mentioned above. The clustering coefficient 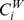 of a node 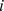 in a weighted network is calculated as [29]:
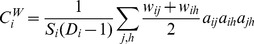
(4)where the normalizing factor 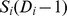 assures that 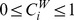; 
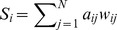
; 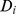 is the degree of a node 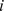. 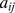 is the connection status between node 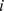 and node 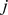. The value of 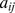 is 1 if there is a link connecting node 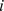 and node 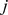, and it is equal to 0 if no connection is presented. This applies to 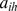 and 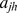 as well. Therefore, the clustering coefficient of a 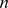-nodes network is calculated as [20]:
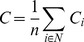
(5)where 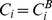 for binary networks and 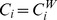 for weighted networks.

The shortest path length 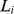 is given by the shortest distance from the node 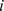 to another node. The shortest path between two nodes could consist of multiple in-between connections when there is no direct connection between them. In comparison to regular networks, complex and random networks generally have short path lengths [26]. The definition of complex, random and regular networks can be found in [4]. The mean path length for a node 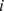 is defined as [20]:
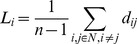
(6)where 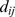 is the shortest distance between node 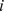 and node 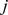. In a binary network, the value of every existing link is 1. 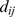 is thus the number of links connecting node 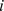 and node 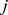. However, for a weighted network, the shortest path length is not necessarily the optimal value, as the weighted network also contains information about connection strength (thickness of link) between nodes [20]. To differentiate the strength of these connections in a weighted network, the strength of every link between node 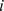 and node 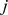 is associated with weight indices 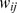. This weight index value was normalized to a range from 0 to 1 [29]. To calculate the weight indices in a weighted network, we followed the approach given by Boccaletti et al. [30]. Let the length between nodes 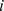 and 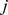 be inversely proportional to the weight indices 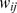:



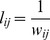
(7)For the weighted network, 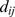 = 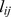. Then the mean shortest absolute path length of the network is the average of shortest absolute path length of all nodes [20]:
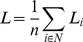
(8)


The global efficiency of a network, 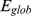, measures the average inverse shortest path length [31]. It is inversely related to the characteristic path length, and it is an alternative way to indicate the parallel information transfer efficiency in the network [4], [32]. It can also be used to describe the connectivity of the network [32], [33]. In comparison to the characteristic path length, the global efficiency makes quantifying disconnected networks possible [20]. Mathematically, for both binary and weighted functional networks, the global efficiency for a node 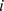 is calculated as [29]:
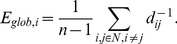
(9)


In comparison to the mean path length (eqn. 6), the global efficiency of a node 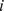 calculates the inverse of the harmonic mean of the minimum absolute path length between node 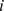 and others [32]. The global efficiency of the network is the average of global efficiency for all nodes and is calculated as:
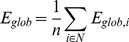
(10)


For binary networks, the local efficiency of the 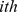 node is calculated as:
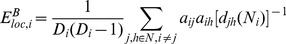
(11)where 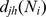 is the shortest path length between 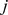 and 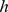 that contains only neighbors of 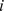. For weighted networks, the local efficiency of the node 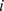 is defined as:
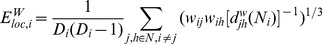
(12)


#### Analysis of Whole-Brain Network Small-World Attributes

Small-world measurements (e.g., [4]) involve a mean cluster coefficient 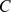 and a mean characteristic path length 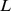. To be specific, the parameter 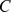 is the average of the clustering coefficient over all nodes in the functional network. It quantifies the level of cliquishness (local interconnectivity) of a typical neighborhood [4]. The parameter 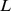 of a network is reflected by the harmonic mean distance between pairs proposed by [34], which is defined as the reciprocal of the average of the reciprocals:
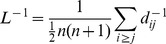
(13)


A high clustering coefficient and a short characteristic path length suggests the network is described by optimal small-world attributes [4], [33], [35]. In other words, a network has less than optimal organization if the absolute path length is relatively short and the absolute clustering coefficient is relatively low [19]. Mathematically, a network would be classified as a small-world network if it satisfies the following two conditions [3]:
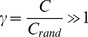
(14)and
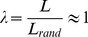
(15)in which 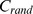 indicates the mean clustering coefficient of a random network and 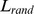 indicates the mean characteristic path length of a random network. The random network preserves the same amount of nodes, links and degree distribution as the functional network. The 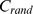 and 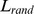 values are calculated by generating many random networks for each individual's functional network. Note that the small-worldness parameter might vary with the change of the sparsity threshold value. When a more rigorous sparsity threshold is chosen, fewer connections will likely exist, leading to a sparser network [36]. Mathematically, the small-worldness is calculated as:
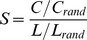
(16)


#### Analysis of Whole-Brain Network Hierarchy

In addition to small-world attributes, the hierarchy was used to characterize topological properties of the human brain [37], as it offered an alternative view on the topological properties of complex networks [38]. The hierarchy of the networks was interpreted by the coefficient 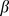, which described the relationship between clustering coefficient 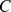 and node degree 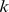 of the network [38] using a power law approach: 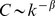. Networks with a high hierarchy value are characterized by a higher degree 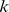 and low clustering coefficient 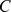, and vice versa. The networks with hierarchical structures contain interconnected clusters, which are the combination of smaller and more densely connected clusters [38].

### Construction of Functional Connectivity Networks

Functional connectivity networks share various significant common ground with anatomical and structural connectivity networks [39], but they also have obvious differences. For example, in structural connectivity networks, connection weights indicate the amount of fibers between regions, the degree of myelination, the probability of connection between two nodes, or the amount of dye that traverse between two nodes, while in functional connectivity studies weights indicate the correlation in the time course of signals of different nodes [5].

**Figure 1:**
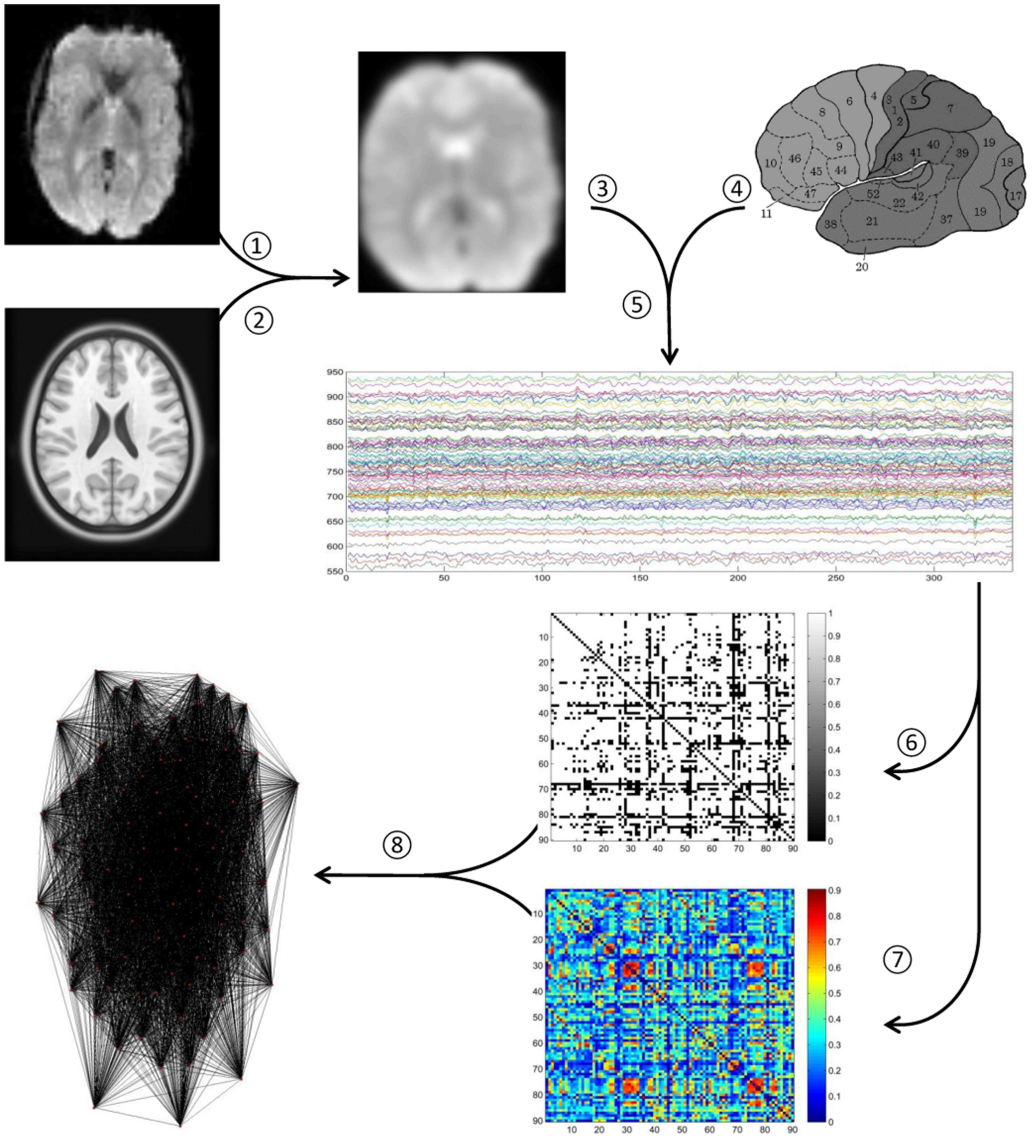
A flowchart for yielding brain connectivity data and network starts with functional (1) and anatomic (2) magnetic resonance imaging scans. In order to establish functional connectivity, a time series of brain activity in different voxels or regions can be derived. These images were later warped to the template (3) to register the location of brain regions. Once scans were registered, the brain regions were parcellated (4) according to the anatomical parcellation scheme described in [24] and 90 regional time series were extracted (5). In order to establish functional connectivity, time series of each brain region were derived and correlations between the time series of different voxels or brain regions were calculated and represented as a correlation matrix. The correlation matrix can be either directly interpreted as a binary network (6) or the weighted network (7). The weighted and binary network can be graphically represented by 3-dimensional connectivity network (8).

Partial correlation could measure the inter-regional functional connectivity by attenuating the contribution of other sources of covariance [40]. A partial correlation matrix is a symmetrical matrix derived from the fMRI time series of each participant. In the correlation matrix, each off-diagonal entry is the correlation between a pair of variables (brain regions) while attenuating their correlation with other variables [19]. In this case, given 90 regions defined in the study in Table 1, a symmetric partial correlation matrix of 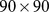 was obtained for each subject. Correlation between any two regions of interest reduced the indirect dependencies of the other 88 regions. When the time series of two brain regions are highly correlated, it implies that the two regions are active at the same time. Using this approach, the mean correlation matrix for all subjects was computed. A sample processing procedure is shown in Figure 1.

**Figure 2:**
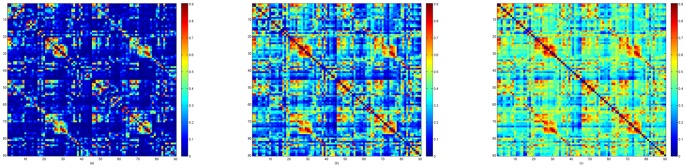
The effects of maintaining different node degrees on the connectivity matrix: (a) 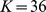; (b) 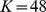; and (c) 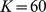.

The individual partial correlation matrices were thresholded to ensure that each node in the network is not too densely clustered, nor too sparsely connected. In other words, thresholding was used in the study to eliminate the links that were likely to attenuate the effect of important connections [20]. The selection of threshold values significantly affected the topological properties of the thresholded networks, as a different number of links in functional networks may represent a different magnitude of correlational interactions. Therefore, to ensure that the partial correlation matrix for each subject had the same number of links, we followed the method proposed by Supekar et al. [33]. Individual partial correlation matrices were thresholded such that each network after thresholding had on average 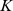 links per node. This approach ensured that both groups had the same number of links per node so that the topological properties of the networks were consistent. Moreover, we selected a conservative 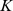 to prevent the generated network from disconnecting or containing non-significant connections. As shown in Figure 2, selecting 60 edges per node produced excessive connections, while selecting 36 edges per node lost important connectivity information. Therefore, as suggested in [33], [41], we selected a 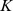 value equal to 48. All networks constructed according to this approach had 2160 edges ( = 48
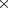
90/2).

To understand the small-world properties of the obtained networks, the value of 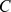 and 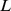 from the functional network were compared with those of 1000 random networks generated by a Markov-chain algorithm [38]. In the random matrix generated by Markov-chain algorithm, if node 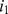 was linked to 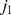 and node 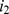 was linked to 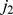, then the link between node 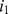 and 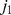 was removed while a link between node 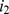 and 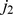 was added [42]. Then the matrix was randomly permuted such that the random matrix and original matrix had equivalent node degree. We repeated this procedure over 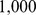 random matrix generated by Markov-chain algorithm to obtain mean 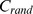 and mean 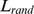 values for every degree and threshold value. In order to study the influence of thresholding, we calculated several network properties as a function of the sparsity thresholds. In order to calculate 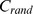 and 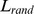, we followed the methodology outlined in [19].

In our study, we examined hierarchy values derived from both whole-brain functional networks and also swallowing related regions. These two connectivity matrices were constructed by thresholding the correlation matrix such that each node in the resulting network generally had 48 connections. The threshold values ranged from 0 to 1, with an increment of 0.05. In order to calculate hierarchy, the clustering coefficient 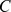 and node degree 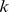 had to be computed for every node in the network. In order to model the relationship between 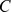 and 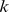, we fitted a fifth order linear regression curve to express the relationship between 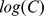 and 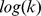.

#### Comparison Between the Whole Brain and Swallowing-Related Regions

In our analysis, we compared the network measures calculated for the whole brain and for the previously identified regions activated during swallowing (e.g., [7], [12], [13], [15]), which are listed in Table 2. We examined whether these network measures were affected by the selected regions.

**Table 2:** Regions of brain activation associated with voluntary saliva swallowing.

Structure	Hemisphere	Structure	Hemisphere
Anterior cingulate and paracingulate gyri	LH/RH	Paracentral lobule	LH/RH
Median cingulate and paracingulate gyri	LH/RH	Inferior parietal, but supramarginal and angular gyri	LH/RH
Posterior cingulate gyrus	LH/RH	Superior parietal gyrus	LH/RH
Cuneus	LH/RH	Postcentral gyrus	LH
Middle frontal gyrus	LH/RH	Precentral gyrus	RH
Superior frontal gyrus, dorsolateral	LH/RH	Precuneus	LH/RH
Fusiform gyrus	LH	Lenticular nucleus, putamen	LH
Hippocampus	LH/RH	Supplementary motor area	LH/RH
Insula	LH/RH	Supramarginal gyrus	LH/RH
Lingual gyrus	LH/RH	Superior tempotal gyrus	LH/RH
Middle occipital gyrus	LH/RH	Thalamus	LH/RH
Superior occipital gyrus	LH/RH		

LH: Left Hemisphere. RH: Right Hemisphere.

#### Network Toolboxes

In this study, we used an open source Brain Connectivity Toolbox (BCT) [20] for calculation of various network properties. The toolbox provided functions for a number of network measures. In addition, the toolbox enabled the network manipulation such as thresholding.

#### Statistical Tests

To distinguish the difference between swallowing related regions to whole brain metrics we used the non-parametric Mann-Whitney Wilcoxon rank-sum test [43].

## Results

Binary and weighted functional networks were created for all subjects using the outlined approach. These functional networks were sensitive to threshold values as shown in Figure 2, which depicted the effects of thresholding the partial correlation matrices such that each node in the resultant network had on average 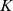 connections. A summary of our results can be found below.

### Network Features

**Figure 3:**
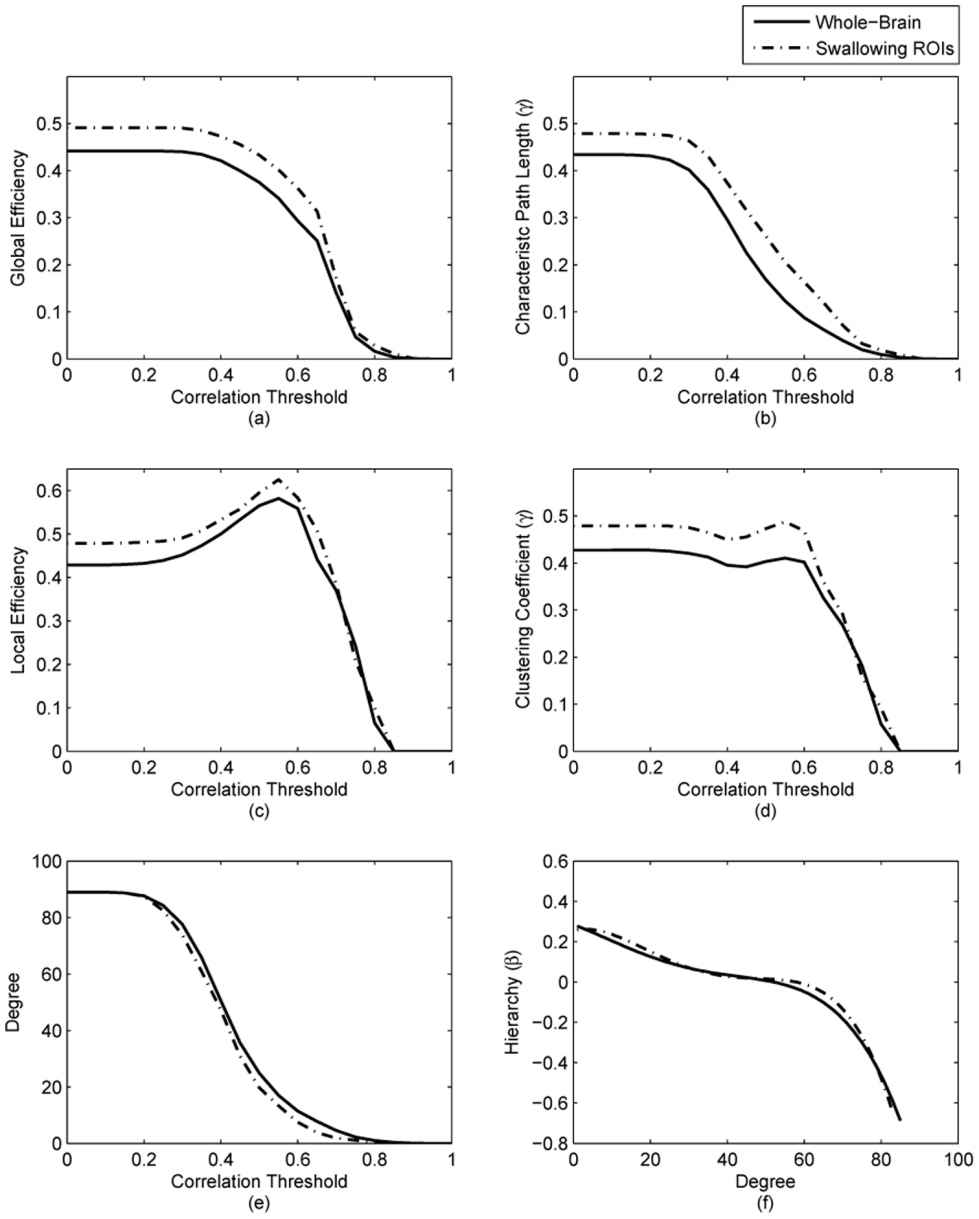
Comparison of networks measures for the swallowing ROIs and the whole brain: (a) global efficiency 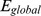 (b) characteristic path length 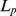 (c) node degree 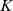 (d) clustering coefficient 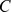 (e) mean local efficiency 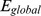 (f) hierarchy 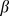.

Figure 3 demonstrated significant differences between the whole-brain matrices and swallowing ROIs for some of the network properties. No obvious difference in node degree was discovered between the two groups (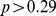). However, global efficiency was higher when considering swallowing ROIs and sparsity threshold values lower than 0.35, but it did not reach statistical significance for all values (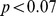). The path length 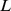 of the binary and weighted network were significantly shorter in the whole-brain metric compared to swallowing related regions (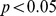) when the threshold value was within the range of 0.60 to 0.85. The local efficiency values were significantly higher when considering swallowing ROIs and threshold values within the range of 0 to 0.03 (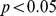). Interestingly, we found that clustering coefficient value has slightly increased when we applied thresholds between 0.5 to 0.63. The rank-sum test showed that significant differences (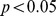) had been found when comparing the whole brain and swallowing ROIs. The observed differences in the clustering coefficient were even greater in this interval in comparison to low threshold values. This has never been found in other network measurement parameters. As shown in Figure 3 (f), the hierarchy values for swallowing ROIs and the whole brain were not statistically different (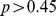).

Our study demonstrated the brain functional networks are characterized by small-world attributes. First of all, the mean network clustering coefficient 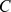 calculated was 0.45 and the mean minimum path length 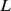 was 0.32. Second, the parameters 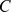 and 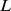 for a random graph with same number of nodes, links and degree distributions were also calculated, and the values were 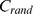 = 0.0116 and 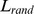 = 0.0119. From the above calculation, we observed that the ratio of local clustering of connections in the brain functional network over the random network was approximately 40 ( 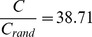 ); whereas, the ratio of path length between any two brain regions was approximately 25 ( 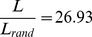 ).

### Inter-Regional Functional Connectivity

**Figure 4:**
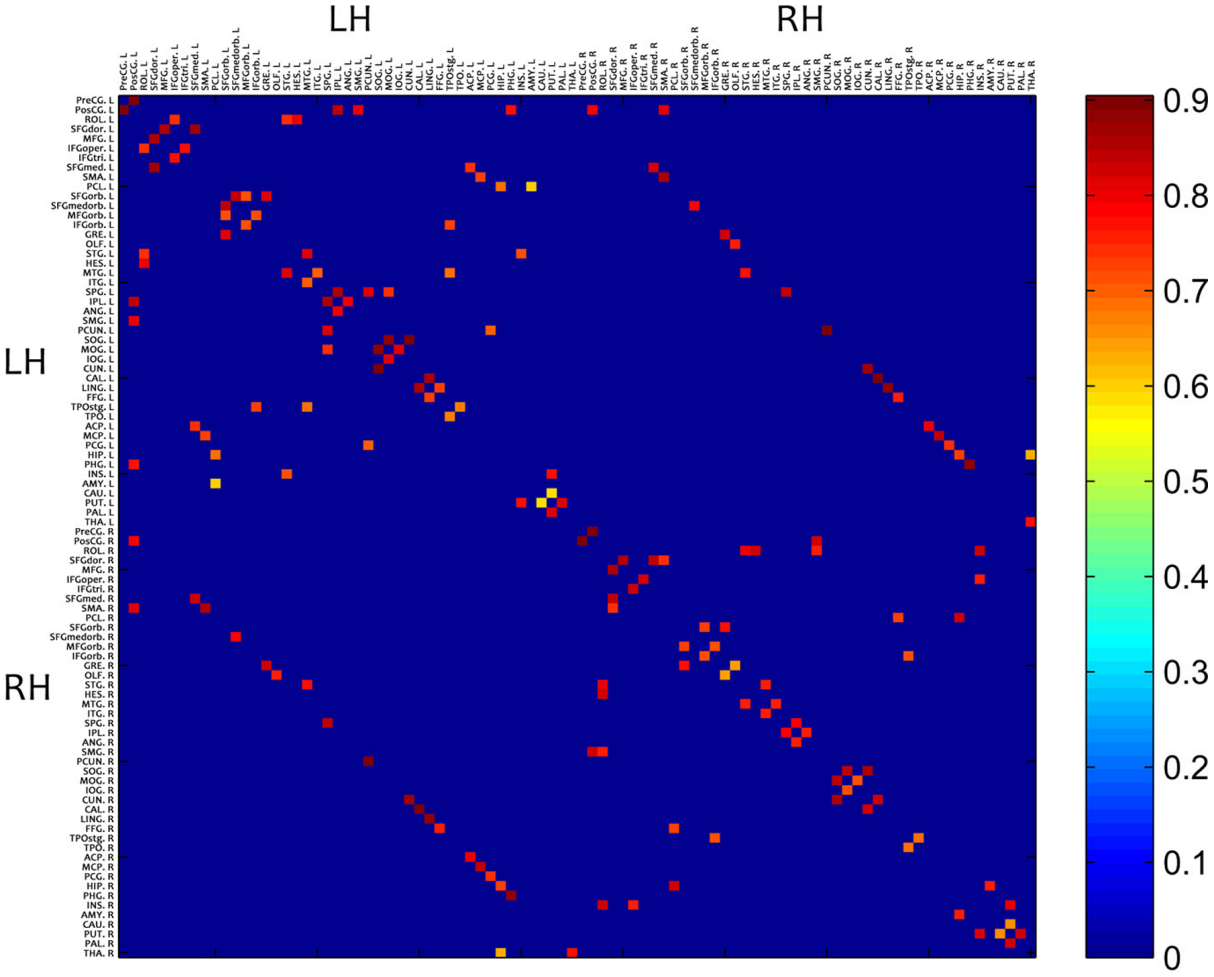
Mean map of the weighted connectivity matrixes averaged across the 22 subjects. LH: Left Hemisphere. RH: Right Hemisphere.

Figure 4 showed the mean inter-regional functional connectivity map. It was derived by averaging across the weighted connectivity matrices of all 22 subjects. The map is a 90 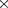 90 symmetric matrix. These 90 regions were classified into six major locations as suggested by Salvador et al. [22]. Each entry in the map represented the percentage of the connectivity strength between the corresponding pair of regions. The value of each entry ranged from 0 (deep blue color in the map) to 1 (dark red color in the map), whereas 0 means no connection at all and 1 means that two corresponding regions were firmly connected.

As we can see in Figure 4, a lot of the connections were long-distance inter-hemispheric connections between bilaterally homologous brain regions. The uniqueness and importance of bilaterally symmetric inter-hemispheric connections can be highlighted in the study of functional network. One reason being that previous multivariate-analyses based brain anatomical network studies were uni-hemispheric, it limits the connections only within a single hemisphere, which were inter-regional connections within left or right hemisphere [22].

## Discussion

We believe that our study is the first one to use novel graph theoretical approaches to report brain functional connectivity during voluntary saliva swallowing. By utilizing the graph theoretical approaches, we are able to study the alteration of functional connectivity at both the global scale as well as the divisional scale.

Our results highlighted that the spatial topological connectivity in swallowing related regions are significantly distinguished compared to whole-brain properties, as can be reflected on various network measurement parameters. Furthermore, our results reported the advantage of applying functional connectivity analysis rather than anatomical connectivity analysis, which is the importance of bilaterally symmetric inter-hemispheric connections. This finding from functional connectivity during swallowing tasks has not been clearly demonstrated by previous studies using anatomical connectivity approaches.

### Network Measures

Network measures for weighted networks in this study consisted of characteristic path length (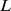), local efficiency (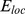), global efficiency (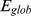), clustering coefficient (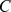), node degree (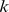), hierarchy (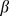), as well as the small-world attributes of the network (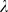 and 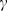). The average value of these network properties across all the 22 subjects were demonstrated in Figure 3. Also, small-world properties, although varying in some degree, were generally found in the weighted networks of every subject in the study. The small-world attributes and hierarchical organization for whole-brain and swallowing ROIs were similar. However, global efficiency, characteristic path length, clustering coefficient and local efficiency shows higher value within the swallowing ROIs in comparison to the whole brain.

The characteristic path length was short in both whole-brain matrices and swallowing-related regions, which indicates that the distance between distinct brain regions are short during swallowing. Although both whole-brain matrices and swallowing-related regions were showing low values, significant differences between these two groups were observed. We have observed that during swallowing the path lengths were significantly different in threshold interval from 0.60 to 0.85, which may suggest the threshold range to use when solely comparing characteristic path length for two different groups. The whole brain had a lower path length than swallowing related regions. This finding suggested that the entire brain functional network during swallowing consists of various short paths between nodes, which provides faster information transfer routes.

A clustering coefficient is defined as the proportion of the number of established connections in direct neighbors of the node to all their possible connections [5]. It can also denote the local efficiency of a network or the network's fault-tolerance [35]. Our study found that the whole-brain values were lower in comparison to the values obtained for the swallowing related regions. To be more specific, we showed that the most significant differences were observed between threshold values 0.5 and 0.63 suggesting that more information was interpreted during swallowing.

Our study also reported small global efficiency values (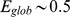) compared to the random network (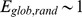); although compared to other network measurements, the difference was not as pronounced between two groups. The smaller 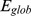 values in functional brain networks compared to random networks showed that the functional brain networks are characterized by small-world properties indicated by [4]. In addition, higher global efficiency values in swallowing-related regions suggest optimal information transfer efficiency of swallowing-related regions in comparison to the whole brain.

### Small-Worldness

Our study revealed that the brain functional network associated with swallowing is a large complex network with efficient small-world properties. The small-world parameters calculated for this study were consistent with small-world attributes for brain functional networks. This further implies that distinct small-world properties are generally found in the weighted networks of every subject in the study. As we have calculated, the clustering coefficient in the brain network was generally 40 times larger than in the random network. That is to say, the brain network is about forty times as clustered when compared to a random network. Also, between any two brain regions in the network, the path length was approximately twenty times longer compared to the random network. A higher absolute clustering coefficient and shorter absolute path length in the functional brain network suggests an optimal small-world profile [41], which benefits the local segregation and global integration within the brain functional network [19].

### Inter-Regional Functional Connectivity

The average functional brain network, shown in Figure 4, primarily consisted of strong connections between closely neighboring brain regions. This demonstrated that anatomically related regions are also likely to be functionally connected. However, functionally connected regions do not necessarily have anatomical connections. Other than intra-hemispheric connections, our data highlighted the bilaterally homologous long-range connections (e.g. PHG.L to PHG.R, SFGmed.L to SFGmed.R, SMA.L to SMA.R and etc). These inter-hemispheric connections were strong in connectivity strength (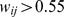) and have not been previously reported according to their anatomical distances [29], which clearly showed the advantage of performing functional network analysis on human brain networks. The importance of bilaterally symmetric inter-hemispheric connections can be highlighted in the study of functional networks. One reason being that previous anatomical connectivity studies on which multivariate analyses have been based were uni-hemispheric; it summarizes inter-regional connections only within a single (right or left) hemisphere [22]. In addition to inter-hemispheric homologus connections, our results demonstrated few non-symmetrical bilaterally inter-hemispheric connections that also have not been reported before, such as SMA.R to PosCG.L, STG.R to HES.L, etc, as shown in Figure 4. These connections were strongly correlated (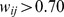) during swallowing tasks.

Compare to previous functional network studies on various tasks, the functional networks during swallowing shows some unique connections. Wang et al. [44] performed functional connectivity analysis during memory encoding and recognition tasks. Their study showed strong functional connectivity between anatomical adjacent regions. However, the bilaterally homologous long-range connections show relatively low connectivity strength (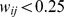), and the unique connections (PosCG.L to SMA.R, HIP.L to THA.R) did not exist in this study. We also referred to other functional connectivity studies [33], [41], and neither of the studies has shown bilaterally homologous long-range connections, which further convinced us of the unique connectivity pattern during swallowing.

Also, the higher degree and stronger strength of functional connectivity in swallowing ROIs (as can be seen in Figure 4) not only demonstrated a more densely connected network during swallowing, but also indicated an increased activation of functionally related brain regions during swallowing.

Correlation between swallowing-related regions in the functional connectivity matrices suggested that this approach could be helpful in understanding the inner connections among regions during swallowing. This approach can also be used as a visualization tool of functional connectivity.

## Conclusion

In this study, we successfully reconstructed the weighted functional networks during swallowing based on fMRI recordings from 22 subjects. We utilized graph-theoretical approaches to produce a set of measures that quantified properties for swallowing-related ROIs and whole-brain metrics of a brain functional network. The main findings in the study were: (1) Swallowing regions and the whole-brain metrics showed a similar node degree distribution and optimal small-world properties. (2) Swallowing-related areas had distinct inter-regional connectivity patterns. (3) The network properties of large-scale brain connectivity differs significantly between swallowing-related areas and the whole brain. Collectively, these and other findings reported in this study provided new insights into how graph-theoretical approaches can be utilized to describe the brain functional network during swallowing and thus provided new clues for understanding the mechanism of swallowing.

## References

[1] Mbonda E, Claus D, Bonnier C, Evrard P, Gadisseux JF, et al (1995) Prolonged dysphagia caused by congenital pharyngeal dysfunction. The Journal of Pediatrics 126: 923–927. 7776095 10.1016/s0022-3476(95)70209-1

[2] Sejdić E, Steele CM, Chau T (2009) Segmentation of dual-axis swallowing accelerometry signals in healthy subjects with analysis of anthropometric effects on duration of swallowing activities. IEEE Transactions on Biomedical Engineering 56: 1090–1097. 19171514 10.1109/TBME.2008.2010504

[3] Sporns O, Zwi J (2004) The small world of the cerebral cortex. Neuroinformatics 2: 145–162. 15319512 10.1385/NI:2:2:145

[4] Watts DJ, Strogatz SH (1998) Collective dynamics of 'small-world' networks. Nature 393: 440–442. 9623998 10.1038/30918

[5] Kaiser M (2011) A tutorial in connectome analysis: Topological and spatial features of brain networks. NeuroImage 57: 892–907. 21605688 10.1016/j.neuroimage.2011.05.025

[6] Hamdy S, Mikulis DJ, Crawley A, Xue S, Lau H, et al (1999) Cortical activation during human volitional swallowing: an event-related fMRI study. American Journal of Physiology – Gastrointestinal and Liver Physiology 277: G219–1-G225-7. 10.1152/ajpgi.1999.277.1.G219 10409170

[7] Kern MK, Jaradeh S, Arndorfer RC, Shaker R (2001) Cerebral cortical representation of reexive and volitional swallowing in humans. American Journal of Physiology – Gastrointestinal and Liver Physiology 280: G354–1-G360-7. 11171617 10.1152/ajpgi.2001.280.3.G354

[8] Dziewas R, Sörös P, Ishii R, Chau W, Henningsen H, et al (2003) Neuroimaging evidence for cortical involvement in the preparation and in the act of swallowing. NeuroImage 20: 135–144. 14527576 10.1016/s1053-8119(03)00285-4

[9] Martin RE, MacIntosh BJ, Smith RC, Barr AM, Stevens TK, et al (2004) Cerebral areas processing swallowing and tongue movement are overlapping but distinct: A functional magnetic resonance imaging study. Journal of Neurophysiology 92: 2428–2493. 15163677 10.1152/jn.01144.2003

[10] Martin RE, Barr AM, MacIntosh B, Smith RC, Stevens T, et al (2007) Cerebral cortical processing of swallowing in older adults. Experimental Brain Research 176: 12–22. 16896984 10.1007/s00221-006-0592-6

[11] Lowell SY, Poletto CJ, Knorr-Chung BR, Reynolds RC, Simonyan K, et al (2008) Sensory stimulation activates both motor and sensory components of the swallowing system. NeuroImage 42: 285–295. 18515150 10.1016/j.neuroimage.2008.04.234 PMC2556067

[12] Martin RE, Goodyear BG, Gati JS, Menon RS (2001) Cerebral cortical representation of automatic and volitional swallowing in humans. Journal of Neurophysiology 85: 938–950. 11160524 10.1152/jn.2001.85.2.938

[13] Mosier KM, Liu WC, Maldjian JA, Shah R, Modi B (1999) Lateralization of cortical function in swallowing: A functional MR imaging study. American Journal of Neuroradiology 20: 1520–1526. 10512240 PMC7657739

[14] Zald DH, Pardo JV (1999) The functional neuroanatomy of voluntary swallowing. Annals of Neurology 46: 281–286. 10482257

[15] Sörös P, Inamoto Y, Martin RE (2009) Functional brain imaging of swallowing: an activation likelihood estimation meta-analysis. Human Brain Mapping 30: 2426–39. 19107749 10.1002/hbm.20680 PMC6871071

[16] Sörös P, Al-Otaibi F, Wong SWH, Shoemaker JK, Mirsattari SM, et al (2011) Stuttered swallowing: Electric stimulation of the right insula interferes with water swallowing. a case report. BMC Neurology 11: 20. 21294905 10.1186/1471-2377-11-20 PMC3045307

[17] Friston KJ, Ashburner JT, Kiebel SJ, Nichols TE, Penny WD, editors (2006) Statistical Parametric Mapping : The Analysis of Functional Brain Images. Jordan Hill, GBR: Academic Press.

[18] Friston KJ, Frith CD, Frackowiak RSJ, Turner R (1995) Characterizing dynamic brain responses with fMRI: A multivariate approach. NeuroImage 2: 166–172. 9343599 10.1006/nimg.1995.1019

[19] Liu Y, Liang M, Zhou Y, He Y, Hao Y, et al (2008) Disrupted small-world networks in schizophrenia. Brain 131: 945–961. 18299296 10.1093/brain/awn018

[20] Rubinov M, Sporns O (2010) Complex network measures of brain connectivity: Uses and interpretations. NeuroImage 52: 1059–1069. 19819337 10.1016/j.neuroimage.2009.10.003

[21] Achard S, Salvador R, Whitcher B, Suckling J, Bullmore E (2006) A resilient, low-frequency, small-world human brain functional network with highly connected association cortical hubs. The Journal of Neuroscience 26: 63–72. 16399673 10.1523/JNEUROSCI.3874-05.2006 PMC6674299

[22] Salvador R, Suckling J, Coleman MR, Pickard JD, Menon D, et al (2005) Neurophysiological architecture of functional magnetic resonance images of human brain. Cerebral Cortex 15: 1332–1342. 15635061 10.1093/cercor/bhi016

[23] Zeng LL, Shen H, Liu L, Wang L, Li B, et al (2012) Identifying major depression using whole-brain functional connectivity: a multivariate pattern analysis. Brain 135: 1498–1507. 22418737 10.1093/brain/aws059

[24] Tzourio-Mazoyer N, Landeau B, Papathanassiou D, Crivello F, Etard O, et al (2002) Automated anatomical labeling of activations in SPM using a macroscopic anatomical parcellation of the MNI MRI single-subject brain. NeuroImage 15: 273–289. 11771995 10.1006/nimg.2001.0978

[25] Brett M, Anton JL, Valabregue R, Poline JB (2002) Region of interest analysis using an SPM toolbox. In: 8th International Conference on Functional Mapping of the Human Brain. Sendai, Japan.

[26] Bullmore E, Sporns O (2009) Complex brain networks: graph theoretical analysis of structural and functional systems. Nature Reviews Neuroscience 10: 186–198. 19190637 10.1038/nrn2575

[27] Barabasi AL, Albert R, Jeong H (2000) Scale-free characteristics of random networks: the topology of the world-wide web. Physica A: Statistical Mechanics and its Applications 281: 69–77.

[28] Sporns O, Chialvo DR, Kaiser M, Hilgetag CC (2004) Organization, development and function of complex brain networks. Trends in Cognitive Sciences 8: 418–425. 15350243 10.1016/j.tics.2004.07.008

[29] Li Y, Liu Y, Li J, Qin W, Li K, et al (2009) Brain anatomical network and intelligence. PLOS Computational Biology 5: e1000395–1-17. 19492086 10.1371/journal.pcbi.1000395 PMC2683575

[30] Boccaletti S, Latora V, Moreno Y, Chavez M, Hwang DU (2006) Complex networks: Structure and dynamics. Physics Reports 424: 175–308.

[31] Latora V, Marchiori M (2001) Efficient behavior of small-world networks. Physical Review Letters 87: 198701–1-4. 11690461 10.1103/PhysRevLett.87.198701

[32] Achard S, Bullmore E (2007) Efficiency and cost of economical brain functional networks. PLoS Computational Biology 3: e17–0174-0183. 17274684 10.1371/journal.pcbi.0030017 PMC1794324

[33] Supekar K, Musen M, Menon V (2009) Development of large-scale functional brain networks in children. PLoS Biology 7: e1000157–1-15. 19621066 10.1371/journal.pbio.1000157 PMC2705656

[34] Newman MEJ (2003) The structure and function of complex networks. SIAM Review 45: 167–256.

[35] Strogatz SH (2001) Exploring complex networks. Nature 410: 268–276. 11258382 10.1038/35065725

[36] Gong G, He Y, Concha L, Lebel C, Gross DW, et al (2009) Mapping anatomical connectivity patterns of human cerebral cortex using in vivo diffusion tensor imaging tractography. Cerebral Cortex 19: 524–536. 18567609 10.1093/cercor/bhn102 PMC2722790

[37] Bassett DS, Bullmore E, Verchinski BA, Mattay VS, Weinberger DR, et al (2008) Hierarchical organization of human cortical networks in health and schizophrenia. The Journal of Neuroscience 28: 9239–9248. 18784304 10.1523/JNEUROSCI.1929-08.2008 PMC2878961

[38] Ravasz E, Barabási AL (2003) Hierarchical organization in complex networks. Physical Review E 67: 026112–1-7. 10.1103/PhysRevE.67.026112 12636753

[39] Honey CJ, Sporns O, Cammoun L, Gigandet X, Thiran JP, et al (2009) Predicting human resting-state functional connectivity from structural connectivity. Proceedings of the National Academy of Sciences 106: 2035–2040. 10.1073/pnas.0811168106 PMC2634800 19188601

[40] Whittaker J (1992) Graphical models in applied multivariate statistics. Journal of Classification 9: 159–160.

[41] Supekar K, Menon V, Rubin D, Musen M, Greicius MD (2008) Network analysis of intrinsic functional brain connectivity in Alzheimer's disease. PLoS Computational Biology 4: e1000100–1-11. 18584043 10.1371/journal.pcbi.1000100 PMC2435273

[42] Liao W, Zhang Z, Pan Z, Mantini D, Ding J, et al (2010) Altered functional connectivity and small-world in mesial temporal lobe epilepsy. PloS one 5: e8525. 20072616 10.1371/journal.pone.0008525 PMC2799523

[43] Mann HB, Whitney DR (1947) On a test of whether one of two random variables is stochastically larger than the other. Analysis of Mathematical Statistics 18: 50–60.

[44] Wang L, Li Y, Metzak P, He Y, Woodward TS (2010) Age-related changes in topological patterns of large-scale brain functional networks during memory encoding and recognition. NeuroImage 50: 862–872. 20093190 10.1016/j.neuroimage.2010.01.044

